# Four new species of *Epicephala* Meyrick, 1880 (Lepidoptera, Gracillariidae) associated with two species of *Glochidion* (Phyllanthaceae) from Hainan Island in China

**DOI:** 10.3897/zookeys.508.9479

**Published:** 2015-06-15

**Authors:** Houhun Li, Zhibo Wang, Bingbing Hu

**Affiliations:** 1 College of Life Sciences, Nankai University, Tianjin 300071, P. R. China

**Keywords:** Lepidoptera, Gracillariidae, *Epicephala*, Phyllanthaceae, *Glochidion*, new species, China

## Abstract

Four new *Epicephala* species that feed on the seeds of *Glochidion sphaerogynum* (Phyllanthaceae) from Yinggeling Mountain Nature Reserves in Hainan Province of China are described: *Epicephala domina* **sp. n.**, *Epicephala impolliniferens* **sp. n.**, *Epicephala angustisaccula* **sp. n.** and *Epicephala camurella* **sp. n.** The latter two species are also associated with *Glochidion wrightii*. Photographs of adults and genital structures are provided.

## Introduction

The genus *Epicephala* Meyrick, 1880 consists of 49 described species worldwide, mainly distributed in the Old World, with 15 species occurring in the Australian Region, 28 in the Oriental Region, one in the Palaearctic Region, and six in the Afrotropical Region (Vári 1961; Kuznetzov 1979; Nielsen et al. 1996; De Prins and De Prins 2005, 2014; Zhang et al. 2012b; Li and Yang 2015). In China, thirteen species have been recorded prior to this study (Meyrick 1935; Kendrick 2005; Hu et al. 2011a, b; Zhang et al. 2012b; Li and Yang 2015; Wang and Li 2015; Yang and Li 2015). But there are still a large number of undescribed species of *Epicephala*, especially in tropical areas (Kawakita et al. 2004; Kawakita and Kato 2006, 2009; Hembry et al. 2012, 2013).

**Figures 1–4.**
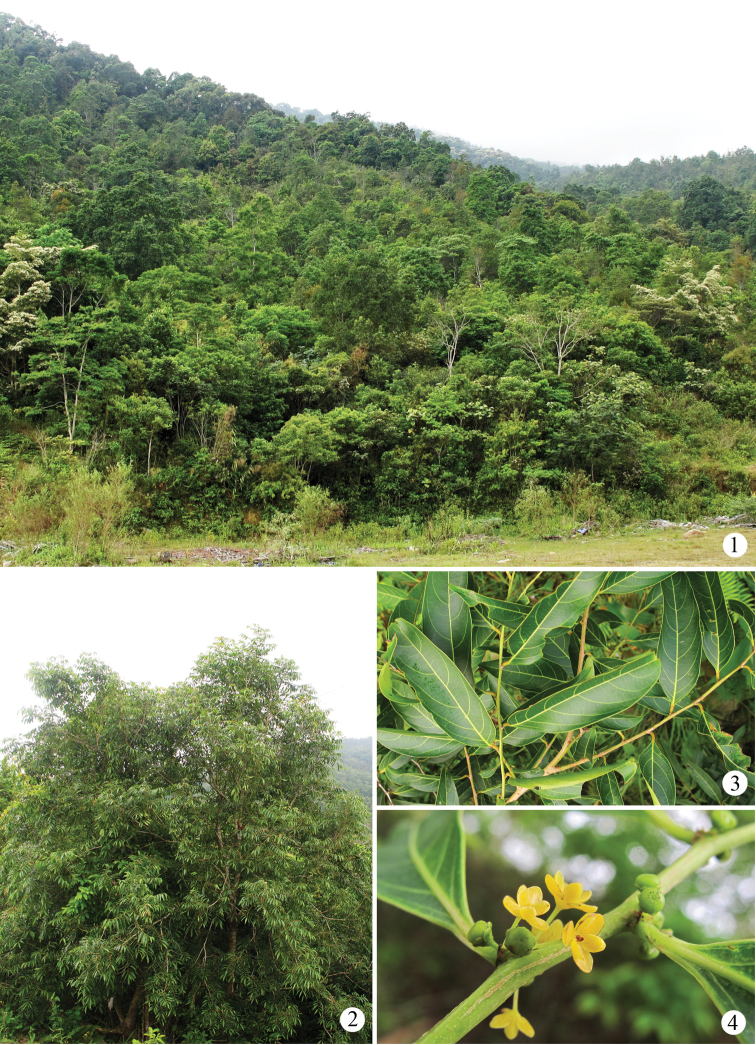
Habitats and common host plant of four *Epicephala* species in Yinggeling Mountain Nature Reserves, Hainan Province, China. **1** general habitat **2–4** morphological features of *Glochidion sphaerogynum*: **2** an individual tree **3** branches and leaves **4** male flowers and young fruits.

In the course of studying the coevolutionary relationships between *Epicephala* moths and *Glochidion* plants in Yinggeling Mountain Nature Reserves of Hainan Province, we identified four new *Epicephala* species. *Epicephala domina* sp. n. and *Epicephala impolliniferens* sp. n. are associated with *Glochidion sphaerogynum* (Müll. Arg.) Kurz; *Epicephala angustisaccula* sp. n. and *Epicephala camurella* sp. n. are associated with both *Glochidion sphaerogynum* (Figs 1–4) and *Glochidion wrightii* Benth.

Larvae of *Epicephala* species feed on seeds in the fruit of their host-plants in the family Phyllanthaceae (Euphorbiaceae *sensu lato*). Some *Epicephala* species have been known to be highly species-speciﬁc with their host-plants within Phyllanthaceae (Kawakita 2010, Hu et al. 2011b, Zhang et al. 2012c), and have presumably coevolved with their hosts. In most cases, within pollinating *Epicephala* and their hosts, a single species of *Epicephala* and a single species of Phyllanthaceae are uniquely associated at a single site, producing so-called “one-to-one” patterns of interaction (Kawakita and Kato 2006; Zhang et al. 2012b). However, in some cases, more complex patterns of specialization, such as one-to-two (Kawakita and Kato 2006; Zhang et al. 2012a), one-to-three (Li and Yang 2015) and two-to-two (Zhang et al. 2012c) patterns, have occurred. We find one-to-four and one-to-two relationships, which will be described further in a separate paper. The present paper just aims at describing the four new *Epicephala* species associated with the two *Glochidion* species from Hainan Island in China.

## Material and methods

Specimens examined in this study were mainly reared from fruits of host-plants, which were gained during a field study from 2009 to 2014 in Yinggeling Mountain Nature Reserves (18°59'30"−19°04'20"N, 109°28'00"−109°35'30"E) in midwestern Hainan Province, China (Fig. 1), and only some were collected on flowers or leaves of two *Glochidion* plants in the late evening. Genitalia dissection and mounting methods follow Li and Zheng (1996). Photos of the host-plant *Glochidion sphaerogynum* were taken in the field using a Canon Power Shot G10 digital camera. Photos of adult specimens were taken with a Leica M250A stereo microscope. Illustrations of the genitalia were prepared by using Leica DM750 microscope, and refined in Photoshop® CS4 software.

The type specimens and vouchers of host plants are deposited in the Insect Collection, College of Life Sciences, Nankai University (NKUM), Tianjin, China and some paratypes are deposited in the Department of Life Sciences, Division of Terrestrial Invertebrates, Natural History Museum, London, UK (BMNH).

## Taxonomic account

### Epicephala domina

Taxon classificationAnimaliaLepidopteraGracillariidae

Li sp. n.

http://zoobank.org/0161B207-D186-47ED-97B6-7FC3518C07E1

**Figures 5–10.**
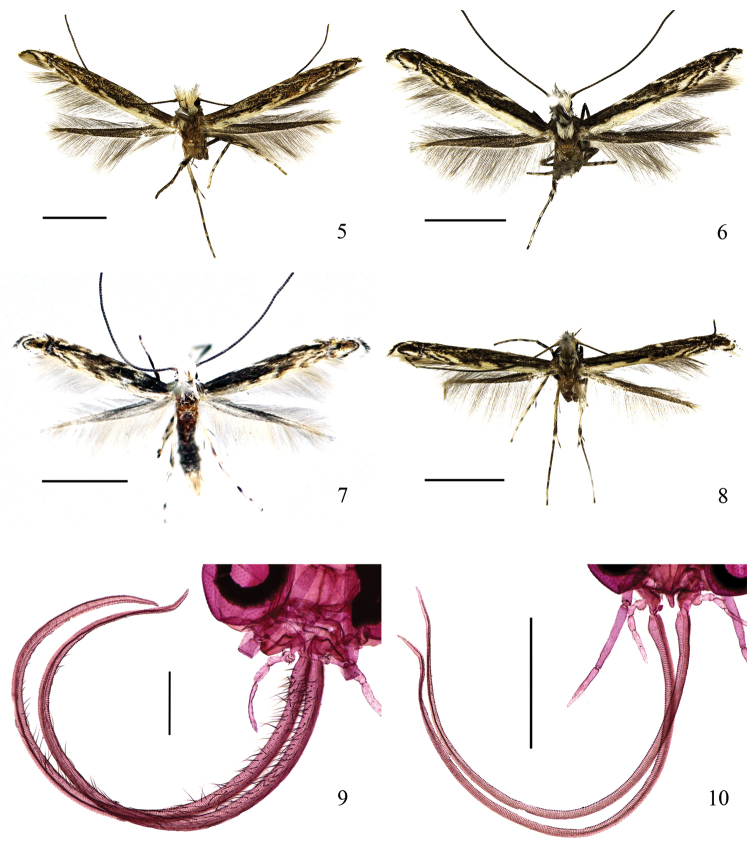
Adult morphology of *Epicephala* spp. **5–8** Adults: **5** *Epicephala domina* sp. n., female paratype **6** *Epicephala impolliniferens* sp. n., female paratype **7** *Epicephala camurella* sp. n., male paratype **8** *Epicephala angustisaccula* sp. n., male paratype **9–10** Morphology of female proboscis: **9** *Epicephala domina* sp. n., female paratype, head slide No. WZB14297 (genitalia slide No. WZB14295 for determination) **10** *Epicephala impolliniferens* sp. n., female paratype, head slide No. WZB14298 (genitalia slide No. WZB14160 for determination). Scale bar: 2.0 mm (**5–6**); 0.5 mm (**9, 10**).

**Figures 11–14.**
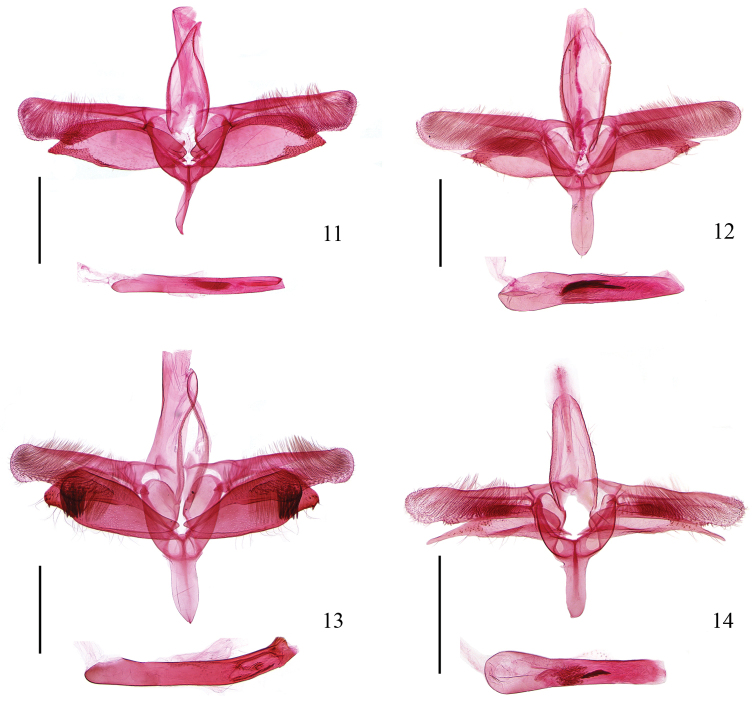
Male genitalia of *Epicephala* spp. **11** *Epicephala domina* sp. n., holotype, genitalia slide No. WZB14337 **12** *Epicephala impolliniferens* sp. n., paratype, genitalia slide No. WZB14278 **13** *Epicephala camurella* sp. n., holotype, genitalia slide No. WZB14043 **14** *Epicephala angustisaccula* sp. n., paratype, genitalia slide No. WZB14001. Scale bar = 0.5 mm.

**Figures 15–16.**
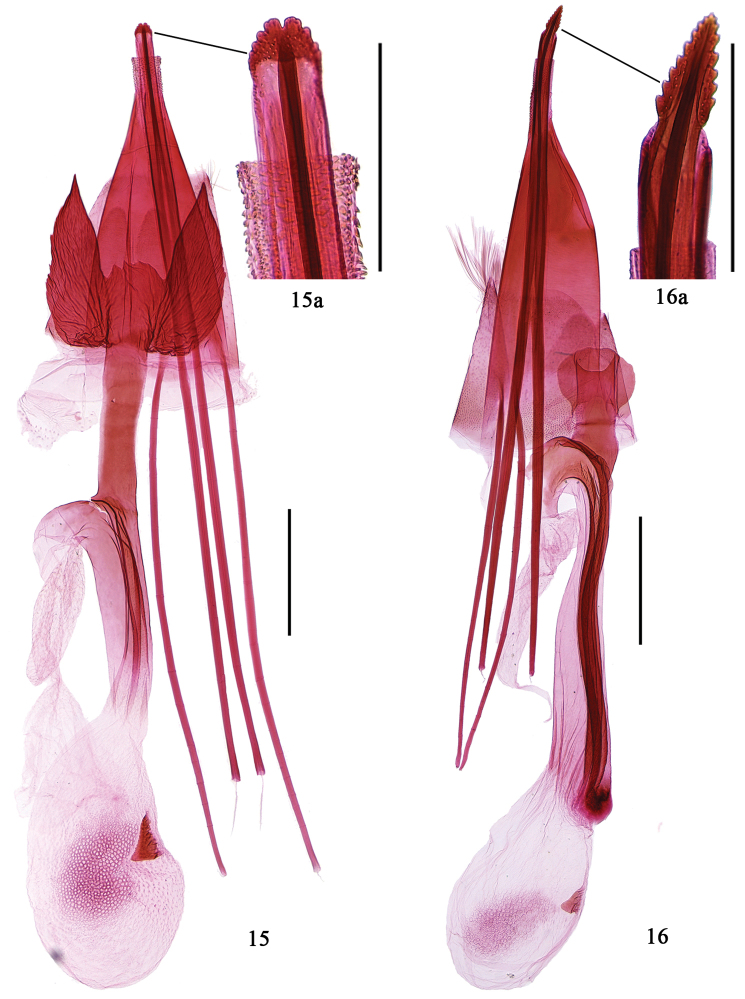
Female genitalia of *Epicephala* spp. **15** *Epicephala domina* sp. n., paratype, genitalia slide No. WZB14295 **16** *Epicephala impolliniferens* sp. n., paratype, genitalia slide No. WZB14249. Scale bar: 0.5 mm (**15, 16**); 0.1 mm (**15a, 16a**).

Figs 5 , 9 , 11 , 15

#### Description.

Adult (Fig. 5). Forewing expanse 7.5−11.0 mm. Head creamy white tinged with pale yellow, lateral sides with long brown scales. Labial palpus white or grayish white, distal half of second and third palpomeres dark brown on outer surface, inner surface of third palpomere white to gray. Antenna grayish brown. Thorax white. Tegula yellowish brown. Forewing grayish brown to deep brown; three pairs white striae from both costal and dorsal margins at 2/5, 3/5 and 4/5 extending obliquely outward to middle as well as to end and outside of cell, third dorsal striae broader and more distinct; dorsal margin with a broad white band extending from base to tornal area; a narrow silvery-white fascia with metallic reflection from costal 6/7 to dorsal margin; distal 1/7 yellowish brown, with a central black dot, with a triangular white dot near costa and a white streak along dorsal margin; cilia basally black, medially grayish white, distally black from distal 1/7 of costal margin along termen to tornus, gray along dorsal margin. Hindwing grayish brown; cilia brown along costal margin and grayish or yellowish brown along dorsal margin.

**Male genitalia** (Fig. 11). Tegumen broadly triangular, narrowed and sclerotized laterally. Tuba analis elongate, gradually narrowed toward apex, exceeding caudal margin of tegumen apically. Costa sub-rectangular, longer than tegumen, distal 1/3 more or less broadened, apex obliquely rounded; dorsal margin straight; ventral margin with basal 3/4 slightly arched inward, with a rounded process at 3/4, with dense long setae on distal half. Sacculus elongate oval, about 4/5 length of costa, acute-angled apically, dorsal margin more arched, distal part of dorsal and ventral margins heavily sclerotized. Transtilla broad at base, long triangular. Vinculum broad V-shaped, rounded on posterior margin; saccus club-shaped, shorter than vinculum, acute at apex. Phallus thin and straight, as long as valva; cornutus in a shape of a rolled plate, with minute spines.

**Female genitalia** (Fig. 15). Ovipositor small, bilobed apically, dentate laterally. Apophysis posterioris slightly longer than apophysis anterioris. Lamella postvaginalis large, as long as 8th abdominal segment, heavily sclerotized, deeply concave at middle on caudal margin to half length, forming two triangles with acute apex. Antrum cylindrical, strongly sclerotized, as long as ductus bursae. Ductus bursae about half length of apophysis anterioris, membranous, with broad longitudinal sclerotized parallel folds, extending from base to corpus bursae; ductus seminalis membranous, arising anterior of antrum. Corpus bursae oval, as long as ductus bursae, with reticulate patches medially; signum a stout tooth, placed at middle.

#### Diagnosis.

This new species is similar to the majority of *Epicephala* species in forewing pattern by having a white dorsal margin, but can be separated from other species by its genitalia. The new species is more similar to *Epicephala ancylopa* Meyrick, 1918, but can be distinguished from the latter in the male genitalia by the ventral margin of the costa with a rounded process at 3/4, the sacculus about 4/5 length of the costa and the acute apex; and in the female genitalia by the lamella postvaginalis as long as the 8th abdominal segment and the presence of signum. In *Epicephala ancylopa* (Lectotype ♂, BMNH, examined; Syntypes: ♂, BMNH, examined, genitalia slide No. 32324, ♀, BMNH, examined, genitalia slide No. 32330, both dissected by Houhun Li), in the male genitalia the ventral margin of the costa has a rounded process at 2/3, the sacculus is about 3/4 length of the costa and bluntly rounded at apex; in the female genitalia the lamella postvaginalis is shorter than the 8th abdominal segment and the signum is absent.

#### Type material.

244♂, 430♀, with genitalia preparations of 244♂ and 93♀.

Holotype ♂ − **CHINA: Hainan Province**: Yinggeling Mountain Nature Reserves (19°01'N, 109°33'E), 450 m, 07.ii.2014, reared from host-plant *Glochidion sphaerogynum* by Zhibo Wang, genitalia slide no. WZB14337.

Paratypes − **CHINA: Hainan Province**: 4♀, Yinggeling Mountain Nature Reserves (19°01'N, 109°33'E), 450 m, 25.xii.2009–25.i.2010, leg. Bingbing Hu; 243♂, 426♀, same locality as holotype, 19.xii.2012–22.i.2013, 12.i.–19.ii.2014, reared or collected from *Glochidion sphaerogynum* by Zhibo Wang (2♂, 2♀, deposited in BMNH).

#### Distribution.

China (Hainan).

#### Biology.

Larvae feed on seeds in the fruits of *Glochidion sphaerogynum* (Müll.Arg.) Kurz (Phyllanthaceae).

#### Etymology.

The specific name is derived from the Latin *dominus* (master, lord), in reference to its status as the dominant *Epicephala* species associated with *Glochidion sphaerogynum*.

### Epicephala impolliniferens

Taxon classificationAnimaliaLepidopteraGracillariidae

Li sp. n.

http://zoobank.org/EBB03B6E-C800-4492-A563-74E31E89EF54

Figs 6 , 10 , 12 , 16

#### Description.

Adult (Fig. 6). Forewing expanse 7.0−10.5 mm. Head white to yellowish white mixed with brown scales. Labial palpus white, inner surface with scattered grayish brown scales; distal 1/2 to 2/3 of second palpomere brown to dark brown on outer surface, third palpomere dark brown on outer surface except tip. Antenna grayish brown to dark brown, each flagellomere paler at base. Thorax white. Tegula dark brown. Forewing brown to dark brown; three pairs white striae from both costal and dorsal margins at 2/5, 2/3 and 3/4 extending obliquely outward to middle as well as to end and outside of cell, second dorsal stria longest and third costal stria shortest; dorsal margin with a broad white band extending from base to tornus; a silvery-white fascia with metallic reflection from costal 6/7 to dorsal margin, gently arched outward; distal 1/7 yellowish brown, with a central black dot, with an indistinct white dot at costa and a white streak along dorsal margin; cilia basally black, medially white, distally black from distal part of costal margin to apex, white from termen to tornus, gray along dorsal margin. Hindwing yellowish white (especially at base) to brown; cilia gray.

**Male genitalia** (Fig. 12). Tegumen broadly oval, sclerotized laterally. Costa as long as tegumen, slightly broadened at base, rounded at apex, with long dense setae ventrally, distal 4/5 nearly parallel dorso-ventrally; dorsal margin straight; ventral margin with lobed process obliquely outward. Sacculus elongate oval, about 3/4 length of costa, acute distally. Transtilla broad at base, narrowed triangularly, curved ventrad distally, acute apically. Vinculum V-shaped, rounded on posterior margin; saccus broad digitiform, as long as vinculum, slightly narrowed at base, rounded at apex. Phallus thick and straight, slightly longer than valva, gently thinned from base to apex; cornuti consisting of four to six spines, usually one or two large, compactly grouped into a bundle.

**Female genitalia** (Fig. 16). Ovipositor small, triangular, dentate laterally, acute apically. Apophysis posterioris obviously thick and strong, 1.5 times longer than apophysis anterioris. Lamella postvaginalis small, rounded, about twice as wide as ostium bursae. Antrum sclerotized, short, as long as lamella postvaginalis. Ductus bursae about same length of apophysis anterioris, membranous, with longitudinal parallel folds, compactly grouped into a broad, heavily sclerotized band extending from base to corpus bursae; ductus seminalis membranous, arising anterior of ductus bursae. Corpus bursae oval, small, about half length of ductus bursae, with reticulate patches medially; signum triangular, placed at middle.

#### Diagnosis.

This species is similar to *Epicephala domina* sp. n. in appearance, but can be separated from the latter by the female proboscis without tip-dilated sensory setae (Fig. 10); in the male genitalia by the apex-rounded costa with a lobed process on ventral margin medially, the sacculus with apex elongate-acute, the phallus with four to six cornuti compactly grouped into a bundle; in the female genitalia by the apically acute ovipositor, the small rounded lamella postvaginalis, the antrum as long as the lamella postvaginalis, the ductus bursae about same length of apophysis anterioris. In *Epicephala domina* sp. n., the female proboscis possesses a large number of tip-dilated sensory setae as most species in the genus (Fig. 9), which can hold numerous pollen grains for pollination; in the male genitalia the costa has an obliquely rounded apex and a rounded protuberance at 3/4 on ventral margin, the sacculus is shortly acute at apex, the cornutus is a rolled plate; in the female genitalia the ovipositor is bilobed at apex, the lamella postvaginalis consists of two triangles with acute apex; the antrum is as long as the ductus bursae, and the ductus bursae is about half length of the apophysis anterioris.

#### Remarks.

*Epicephala impolliniferens* sp. n. is the first species of non-pollinating *Epicephala* associated with *Glochidion*, and the second named species within the genus (following *Epicephala relictella* Kuznetzov, 1979) in which the female proboscis lacks the tip-dilated sensory setae on its surface for carrying pollens. Species of the genus *Epicephala* are noteworthy for their obligate pollination habits, which involve mutualistic relationship with trees of Phyllanthaceae. However, both *Epicephala impolliniferens* sp. n. and *Epicephala relictella* Kuznetzov are not associated with pollination in biology referring to the morphology of the female proboscis. *Epicephala relictella* feeds on the seeds of *Flueggea suffruticosa* (Pall.) Baill. (Hu et al. 2011b). Kawakita and Kato (2009) reported several undescribed *Epicephala* species that do not pollinate their hosts. We have confirmed one of them not belonging to the genus *Epicephala* (unpublished data), and the status of the other undescribed species needs to be determined.

#### Type material.

48♂, 64♀, with genitalia preparations of 48♂ and 46♀.

Holotype ♂ − **CHINA: Hainan Province**: Yinggeling Mountain Nature Reserves (19°01'N, 109°33'E), 450 m, 11.i.2013, reared from host-plant *Glochidion sphaerogynum* by Zhibo Wang, genitalia slide no. WZB14178.

Paratypes − **CHINA: Hainan Province**: 3♂, 5♀, Yinggeling Mountain Nature Reserves (19°01'N, 109°33'E), 450 m, 12.vi.2010, 18–26.ix.2010, leg. Bingbing Hu; 45♂, 58♀, same locality as holotype, 19.xii.2012–24.i.2013, 12.i.–20.ii2014, reared or collected from *Glochidion sphaerogynum* by Zhibo Wang (2♂, 2♀, deposited in BMNH).

#### Distribution.

China (Hainan).

#### Biology.

Larvae feed on seeds in the fruits of *Glochidion sphaerogynum* (Müll. Arg.) Kurz (Phyllanthaceae).

#### Etymology.

The specific name is derived from the Latin *im*- (= not), *pollinicus* (= pollen) and *ferre* (= to carry, to bear), in reference to the non-pollinating habit.

### Epicephala camurella

Taxon classificationAnimaliaLepidopteraGracillariidae

Li sp. n.

http://zoobank.org/55573693-A511-43CD-8250-E553797D20B6

Figs 7 , 13 , 17

#### Description.

Adult (Fig. 7). Forewing expanse 7.0−10.0 mm. Head white, mixed with brown scales. Labial palpus white, second and third palpomeres dark brown on outer surface, second palpomere scattered with grayish brown scales on inner surface. Antenna grayish brown to dark brown. Thorax white. Tegula brown, with a few grayish white or brown mottled white scales distally. Forewing grayish brown to brown, sometimes tinged with ochreous scales; three pairs white striae from both costal and dorsal margins at 1/3, 3/5 and 4/5 extending obliquely outward to middle and end of cell as well as to outside of cell, second dorsal stria longest and extending to 6/7; dorsal margin with a broad white band from base to tornus; a silvery-white fascia with metallic reflection from costal 6/7 to dorsal margin, nearly straight; distal 1/7 ochreous, with a central black dot, with a white dot at costa and a broad white streak along dorsal margin; cilia white from distal part of costal margin along termen to tornus except black at base and apex, grayish white to gray along dorsal margin. Hindwing gray; cilia grayish white to gray.

**Male genitalia** (Fig. 13). Tegumen elongately oval, sclerotized laterally. Tuba analis long and broad, more or less sclerotized, apically far exceeding caudal margin of tegumen. Costa as long as tegumen, subrectangular, slightly narrowed before rounded apex, with long dense setae ventrally; dorsal margin nearly straight; ventral margin sinuate, with a small protuberance at 2/5, with a large roundly protuberance at 3/5, bearing long strong spines distally. Sacculus broad, subtriangular, about 3/4 length of costa, narrowed at base, widened to about middle, distal half parallel except slightly concave ventrally before apex; apex truncate obliquely, with sparse short spines ventrally. Transtilla broad at base, elongate triangular, acute apically. Vinculum V-shaped, rounded on posterior margin; saccus broad digitiform, shorter than vinculum, apex obtusely acute. Phallus long and strong, about 1.5 times longer than valva, gently curved at distal 1/3, with pieces of irregular sclerites in distal 1/3; cornutus absent.

**Figures 17–18.**
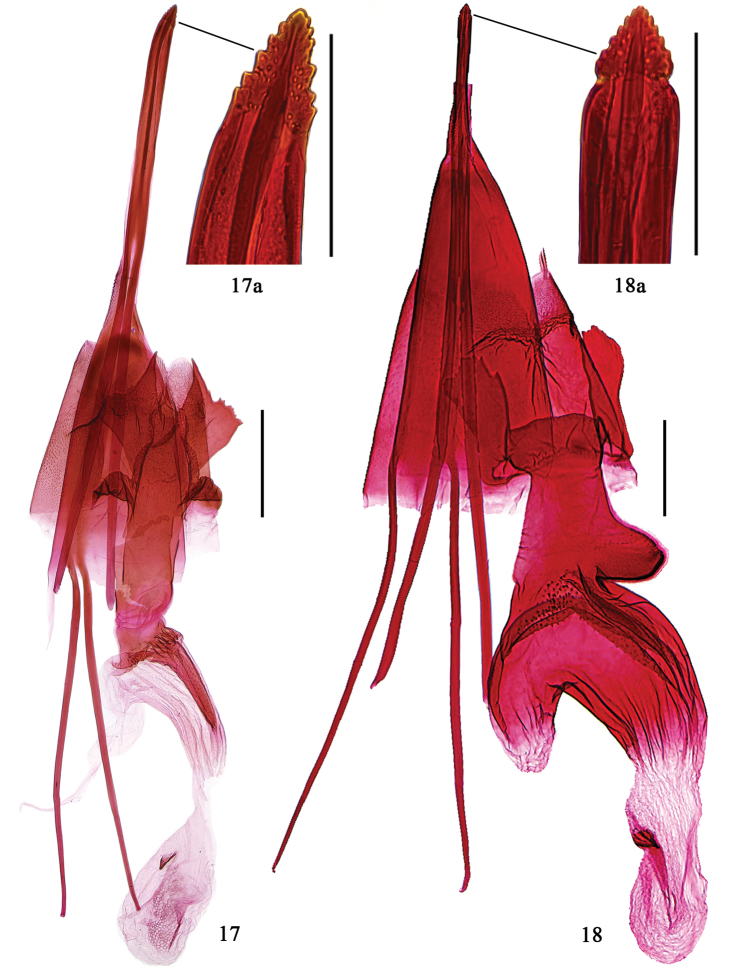
Female genitalia of *Epicephala* spp. **17** *Epicephala camurella* sp. n., paratype, genitalia slide No. WZB14253 **18** *Epicephala angustisaccula* sp. n., paratype, genitalia slide No. WZB14412. Scale bar: 0.5 mm (**17, 18)**; 0.1 mm (**17a, 18a**).

**Female genitalia** (Fig. 17). Ovipositor small, triangular, dentate laterally, acute apically. Apophysis posterioris 1.8 times longer than apophysis anterioris. Lamella postvaginalis developed, heavily sclerotized, deeply concave at middle caudally, forming two rhombic plates originated from caudal margin of ostium bursae, outer margin serrate, apex acute. Lamella antevaginalis a pair of sclerotized and curved carinae. Antrum sclerotized, thick and strong, as long as 8th abdominal segment. Ductus bursae membranous, about same length as antrum, with longitudinal sclerotized folds extending from base to 2/3; ductus seminalis membranous, arising anterior of ductus bursae. Corpus bursae oval, small, about 2/3 length of ductus bursae, with reticulate patches medially; signum triangular, small, placed at posterior 1/3.

#### Diagnosis.

This new species is similar to *Epicephala frenata* Meyrick, 1908, but differs from the latter in the shape of sacculus and phallus in male genitalia as well as in the shape of sterigmatic sclerotizations in female genitalia. In the male genitalia of the new species, the apex of the sacculus is truncate obliquely and the phallus is gently curved at distal 1/3; in the female genitalia, the caudal margin of the lamella postvaginalis is deeply concave medially and the lamella antevaginalis is a pair of sclerotized and curved carinae. In the male genitalia of *Epicephala frenata* (Syntype♂, BMNH, examined, genitalia slide No. 32303, dissected by Houhun Li; syntype♀, BMNH, examined, genitalia slide No. 32304, dissected by Houhun Li), the apex of the sacculus is broadly rounded and the phallus is straight; in the female genitalia, the caudal margin of the lamella postvaginalis is slightly concave and the lamella antevaginalis is absent.

#### Type material.

20♂, 42♀, with genitalia preparations of 20♂ and 42♀.

Holotype ♂ − **CHINA: Hainan Province**: Yinggeling Mountain Nature Reserves (19°01'N, 109°33'E), 450 m, 29.xii.2012, reared from host-plant *Glochidion wrightii* by Zhibo Wang, genitalia slide no. WZB14043.

Paratypes − **CHINA: Hainan Province**: 12♂, 25♀, Yinggeling Mountain Nature Reserves (19°01'N, 109°33'E), 450 m, 06.i.–08.vi.2010, reared or collected from *Glochidion wrightii* by Bingbing Hu; 2♂, same locality as holotype except the dates 11–12.iv.2011, reared from *Glochidion wrightii* by Jing Zhang; 5♂, 15♀, same locality as holotype except the dates 28.xii.2012–24.i.2013, reared from *Glochidion wrightii* by Zhibo Wang; 2♀, same locality except the date 12.i.2013, reared from *Glochidion sphaerogynum* by Zhibo Wang (1♂, 1♀, deposited in BMNH).

#### Distribution.

China (Hainan).

#### Biology.

*Glochidion wrightii* is the primary host-plant and *Glochidion sphaerogynum* (Phyllanthaceae) is secondary. Larvae feed on seeds in the fruit.

#### Etymology.

The specific name is derived from the Latin *camur* (curved) and postfix -*ella*, in reference to the lamella antevaginalis being a pair of sclerotized and curved carinae in the female genitalia.

### Epicephala angustisaccula

Taxon classificationAnimaliaLepidopteraGracillariidae

Li sp. n.

http://zoobank.org/C083610A-8261-4A76-8E1F-D1F7012B2620

Figs 8 , 14 , 18

#### Description.

Adult (Fig. 8). Forewing expanse 7.0−8.5 mm. Head grayish white to white, laterally mixed with brown scales. Labial palpus grayish white, grayish brown on outer surface of second palpomere, basal 2/3 of third palpomere brown. Antenna grayish brown. Thorax dirty white to snowy white; tegula grayish white to brown. Forewing grayish brown to deep brown; costal margin with three parallel white striae obliquely extending outward from basal 1/3, 1/2 and 3/4 respectively, first and third striae broad and short, reaching 1/3 of wing width, second stria narrow and long, reaching midwing; a broad creamy white band extending from base to tornus along dorsal margin, its upper margin extended to a broad, ill-defined white stria at 2/5, reaching below fold dorsally, second white stria from 2/3 obliquely outward to meet second costal stria at midwing, third stria from beyond second one and parallel with it to midwing, sometimes meeting third costal stria; a silvery fascia with metallic reflection from costal 5/6 to dorsal margin, slightly arched outward medially; distal 1/6 yellowish brown, with a central black dot, with a small white dot at costa and a white streak along dorsal margin; cilia white from distal part of costal margin along termen to tornus except black at base and apex, gray along dorsal margin. Hindwing gray to deep gray, sometimes basal 1/3 densely covered with rough black scales; cilia gray.

**Male genitalia** (Fig. 14). Tegumen elongate oval, sclerotized laterally. Costa longer than tegumen, nearly parallel dorso-ventrally, rounded at apex, with long dense setae ventrally; dorsal margin slightly sinuate; ventral margin slightly protruded with strong short setae at 3/4, then concave inward before apex. Sacculus elongate triangular, about 4/5 length of costa, slightly curved ventrad, tapered distally. Transtilla triangular, curved ventrad distally, acute apically. Vinculum broad U-shaped, rounded on posterior margin; saccus broad digitiform, as long as vinculum, apex rounded. Phallus straight, longer than valva, expanded in basal 1/3; cornuti with four spines compactly grouped into a bundle.

**Female genitalia** (Fig. 18). Ovipositor small and short, triangular, dentate laterally, acute apically. Apophysis posterioris 1.6 times longer than apophysis anterioris. Lamella postvaginalis well developed, heavily sclerotized, composed of two narrow, widely spaced rectangular plates derived from caudal margin of ostium bursae, about half length of 8th abdominal segment, caudal margin serrated. Ostium bursae sclerotized, broad. Antrum heavily sclerotized, broad, as long as 8th abdominal segment, with large rounded appendix protruding near ductus bursae on right side. Ductus bursae slightly longer than antrum, sclerotized, expanded, broader than corpus bursae; ductus seminalis arising from base of ductus bursae. Corpus bursae elongate oval, as long as ductus bursae; signum triangular, small, placed at posterior 1/3.

#### Diagnosis.

This species is similar to *Epicephala domina* sp. n. in appearance and genitalia, but can be separated from the latter in the male genitalia by the subtriangular sacculus and the dilated basally phallus; in the female genitalia by the apically acute ovipositor and the lamella postvaginalis being shorter than 8th abdominal segment. In *Epicephala domina* sp. n., in the male genitalia the sacculus is broad-oval and the phallus is not dilated basally; in the female genitalia the ovipositor is bilobed apically and the lamella postvaginalis is as long as 8th abdominal segment.

#### Type material.

5♂, 1♀, with genitalia preparations of 5♂ and 1♀.

Holotype ♂ − **CHINA: Hainan Province**: Yinggeling Mountain Nature Reserves (19°01'N, 109°33'E), 450 m, 18.i.2014, reared from the host-plant *Glochidion sphaerogynum* by Zhibo Wang, genitalia slide no. WZB14001.

Paratypes − **CHINA: Hainan Province**: 1♂, Yinggeling Mountain Nature Reserves (19°01'N, 109°33'E), 450 m, 02.x.2010, reared from *Glochidion sphaerogynum* by Bingbing Hu; 3♂, same locality as holotype except the dates 29.x.2013 and 18–25.i.2014, reared or collected from *Glochidion sphaerogynum* by Zhibo Wang; 1♀, same locality as holotype except the date 15.v.2010, reared from *Glochidion wrightii* by Bingbing Hu; 1♂, same locality as holotype except the date 24.i.2014, reared from *Glochidion wrightii* by Zhibo Wang.

#### Distribution.

China (Hainan).

#### Biology.

Larvae feed on seeds in the fruits of *Glochidion sphaerogynum* (Müll.Arg.) Kurz and *Glochidion wrightii* Benth. (Phyllanthaceae).

#### Etymology.

The specific name is derived from the Latin *angustus* (narrow) and *sacculus*, in reference to the distally narrowed sacculus in the male genitalia.

## Supplementary Material

XML Treatment for Epicephala domina

XML Treatment for Epicephala impolliniferens

XML Treatment for Epicephala camurella

XML Treatment for Epicephala angustisaccula
